# Gold-catalyzed propargylic substitutions: Scope and synthetic developments

**DOI:** 10.3762/bjoc.7.99

**Published:** 2011-06-28

**Authors:** Olivier Debleds, Eric Gayon, Emmanuel Vrancken, Jean-Marc Campagne

**Affiliations:** 1 Institut Charles Gerhardt, UMR 5253, Equipe AM2N, ENSCM 8 rue de l’Ecole Normale, 34296 Montpellier Cédex, France

**Keywords:** direct substitutions, gold, isoxazolines, propargylic substitutions

## Abstract

This personal account summarizes our recent developments in gold-catalyzed direct substitutions on propargylic (allylic, benzylic) alcohols, with various nucleophiles (and bi-nucleophiles) based on the σ- and/or π-acidity of gold(III) complexes. Synthetic developments are also briefly described.

## Introduction

In the field of nucleophilic substitution reactions, leaving groups are mostly often obtained from alcohols but initially require their transformation to better leaving groups such as sulfonates or acetates. For economic, environmental and practical reasons it is therefore of interest to develop new experimental conditions for the direct substitution of activated alcohols such as tertiary, allylic, benzylic or propargylic ones [1–4]. Since some pioneering work using stoichiometric amounts of Lewis acid catalysts [3–5], much effort has been devoted to this goal. In this context, we have been particularly interested in the direct substitution of propargylic alcohols, because i) the presence of the alkyne function in the substitution product allows many further synthetic modifications, ii) the challenge of controlling the possible competition between substitutions at the propargylic and/or the allenic positions [6], and iii) compared to allylic and benzylic substitutions these reactions have been studied to a far lesser extent. Direct propargylic substitutions have traditionally and efficiently been carried out using the Nicholas [7] conditions but this implies the use of stoichiometric amounts of a cobalt complex. In 1994, Murahashi [8] described in a seminal paper the propargylic substitution of monosubstituted alkynes bearing a good leaving group on the propargylic alcohol moiety, where a mechanism through a copper-allenylidene intermediate was postulated. Subsequently, asymmetric versions have been described [9–10]. Thus, Hidai, Nishibayashi and Uemura developed a diruthenium complex catalyst for the direct substitution of monosubstituted propargylic alcohols (R^3^ = H), by a large number of heteroatomic and carbon centered nucleophiles [11–15]. Enantiomerically pure ruthenium catalysts for asymmetric propargylic substitutions were next developed using acetone, hydrides and electron rich aromatics as nucleophiles [16–18]. In 2003, oxo-rhenium catalysts were introduced by Toste [19–21]. Substitution products were obtained in high yields with alcohols, allylsilanes, aromatic compounds and nitrogen nucleophiles. Interestingly, these reactions were not limited to monosubstituted propargylic alcohols [19–22].

In 2005, we described the direct Au(III)-catalyzed substitution of propargylic alcohols in the presence of various nucleophiles (allylsilanes, alcohols, thiols, electron rich aromatic compounds), and showed that gold probably acts as a Lewis acid to promote the formation of a stabilized propargylic carbocation intermediate [23–24]. A related reaction was subsequently reported by Dyker [25] in 2006, using azulene and 1,3-dimethoxybenzene in Friedel–Crafts type reactions with benzylic and propargylic alcohols. Shortly after, Sanz and Zhan discovered, that these reactions could also be carried out under Brønsted acid and FeCl_3_ catalysis, respectively [26–29]. Later, the use of copper, indium, bismuth, scandium, ytterbium, phosphomolybdic acid and iodine catalysts were reported by several groups worldwide [30–39]. These aspects have been recently reviewed [40–43]. Among all these various Lewis acid catalysts, gold stands out since it possesses a unique hard/soft Lewis acid dichotomy allowing the activation of both alcohols and π-bonds. We therefore assumed that we could take advantage of this ambivalence in order to perform new domino processes [44–45]. Moreover, due to these ambivalent properties, the control of the chemo- and regioselectivity is challenging and raises interesting mechanistic considerations.

In this special issue dedicated to "Gold catalysis for organic synthesis", we would like to give a personal account on our work related to this topic: Scope, limitations and synthetic utilization of the gold(III)-catalyzed direct substitution of alcohols and the development of domino reactions.

## Review

### Gold-catalyzed alcohol substitution

#### Scope and limitations

**Scheme 1:**
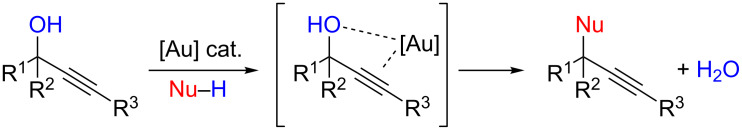
Gold-catalyzed propargylic substitutions.

In the past few years, homogeneous gold catalysis has emerged as an efficient tool to activate triple bonds for the addition of various nucleophiles to alkynes. We initially anticipated that, through coordination to π-bond [46–48], gold catalysts may also act as propargylic alcohol-activating agents in propargylic substitutions (Scheme 1).

**Scheme 2:**
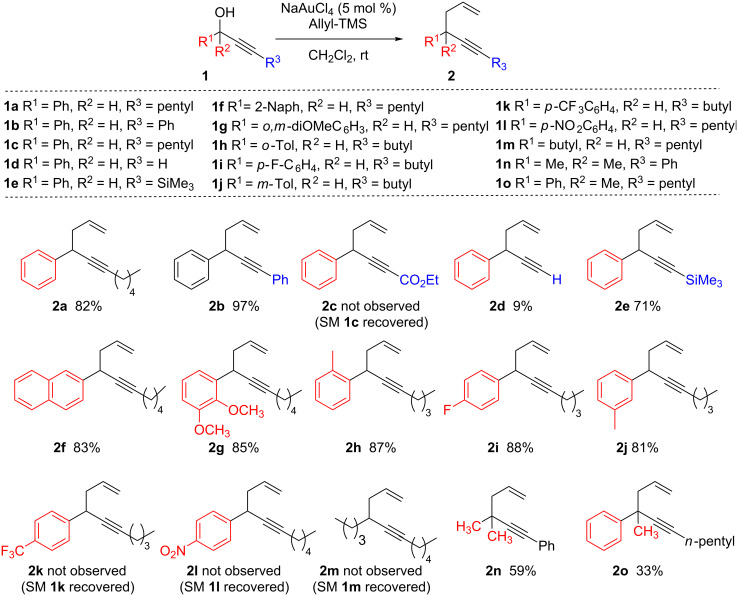
Propargylic substitution: scope of substrates.

To validate this hypothesis, the reactivity of 1-phenyloct-2-yn-1-ol (**1a**, R^1^ = Ph, R^2^ = H, R^3^ = *n*-pentyl) with allyltrimethylsilane in the presence of various gold catalysts at room temperature was investigated (Scheme 2, Table 1). Gratifyingly, the reaction proved to be efficient with various Au(III) reagents (at 5% catalyst loading) (Table 1, entries 1–4). The best results were observed with NaAuCl_4_·2H_2_O (Table 1, entry 1). In the presence of Au(I) catalysts (Table 1, entries 5 and 6), the results were more disappointing. Under the same reaction conditions (dichloromethane at room temperature), no reaction occurred in the presence of PtCl_2_ and PdCl_2_(PhCN)_2_ catalysts.

**Table 1:** Catalyst screening.

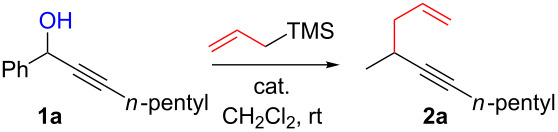			

Entry	Gold cat (%)	Time (h)	**2a** Isolated yield (%)

1	NaAuCl_4_·2H_2_O (5)	12	82
2	AuBr_3_ (5)	12	68
3	AuCl_3_ (5)	12	65
4	HAuCl_4_·3H_2_O (5)	12	60
5	AuCl (10)	12	30
6	Ph_3_PAuCl (10)	12	NR^a^
7	PdCl_2_(PhCN)_2_ (5)	12	NR^a^
8	PtCl_2_(5)	12	NR^a^

^a^NR = no reaction.

Based on the results of these initial experiments, we set out to define the scope of these reactions by first examining variations on propargylic alcohols **1a**–**o**. As illustrated in Scheme 2, the effect of the nature of the substituent on the acetylenic position (R^3^ group, Scheme 2) was examined. The following trend was observed: Ph > alkyl > SiR_3_ > H >> CO_2_Et. Indeed, the best yield (**2b,** 97%) was obtained with propargylic alcohol **1b** bearing a phenyl group and no reaction was observed when an electron-withdrawing group such as ester was present on the propargylic alcohol **1c**. It is worth noting that with terminal alkynes, the product **2d** was obtained in low yield (9%). The presence of various substituted aromatic groups on the propargylic position (R^1^, R^2^ groups), i.e., compounds **1f**–**1j** (for other examples, see reference [24]) gave good product yields, whilst aryl groups with strong electron-withdrawing groups such as *p*-NO_2_ **1l** and *p*-CF_3_ **1k** were unreactive. When the aryl group was replaced by an alkyl chain, no reaction occurred (as illustrated with **1m**) whereas when two alkyl substituents were present, as in **1n**, the reactivity was restored to give **2n** in 59% yield. When both an alkyl and an aromatic group were both present, as in **1o**, a lower yield was obtained due to rapid decomposition of the product **2o** at room temperature. It should be emphasized that all the allyl substituted products **2a**–**2o** have, in general, low stabilities and decompose within a few days at room temperature. Two general conclusions can be drawn from these experiments using allylsilanes as nucleophiles: A stabilization of a positive charge (+ or δ+) on the propargylic position by electron-donating groups on the propargylic and/or acetylenic positions favors the reaction, and no product resulting from attack at the acetylenic position could be observed.

**Scheme 3:**
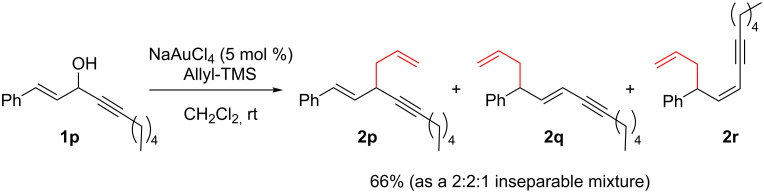
Propargylic substitutions on allylic/propargylic substrates.

To explore further regioselectivity issues, compound **1p**, which is both an allylic and a propargylic alcohol, was submitted to the same reaction conditions. A 2:2:1 inseparable mixture of S_N_ **2p** and S_N_’ **2q** and **2r** products was obtained (Scheme 3).

**Scheme 4:**
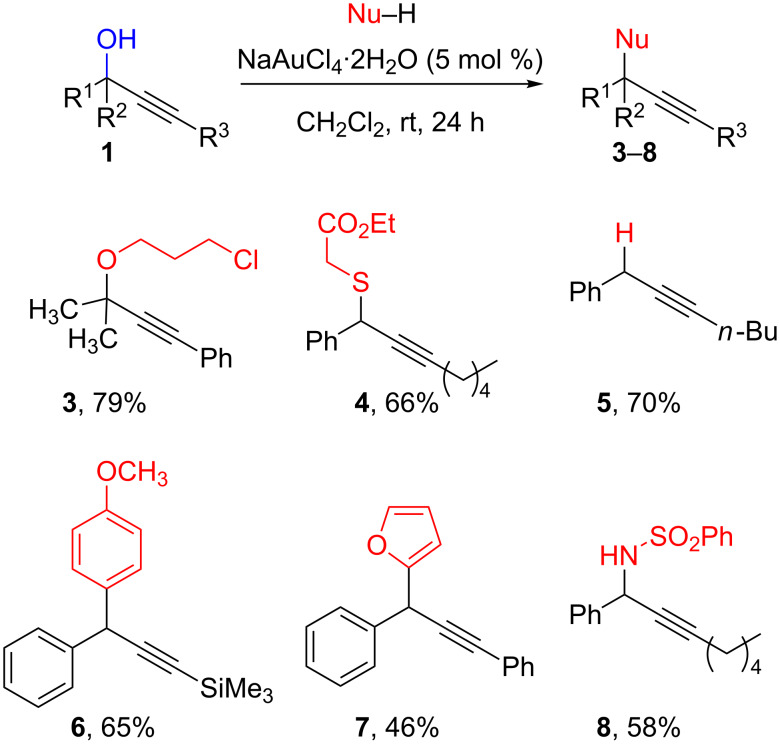
Direct propargylic substitutions: Scope of nucleophiles.

The Au(III)-catalyzed reaction was next investigated for diverse series of nucleophiles. A large number of nucleophiles are very effective in these reactions including alcohols, thiols, hydrides (from Et_3_SiH or PMHS), electron-rich aromatic and hetero-aromatic derivatives such as anisole or furan, and deactivated nitrogen nucleophiles such as phenylsulfonamide (compounds **3**–**8**, Scheme 4).

**Scheme 5:**
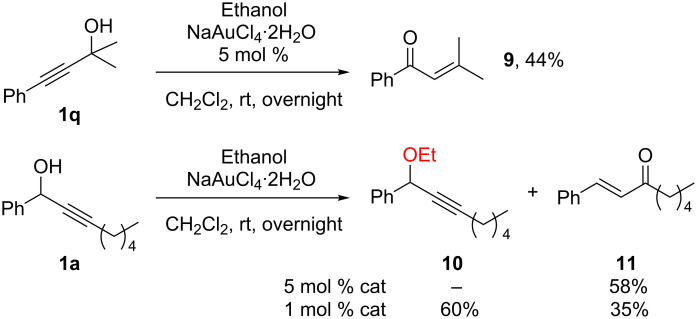
Meyer–Schuster rearrangements.

In some instances unexpected selectivities and further transformations were observed. When ethanol was used as the nucleophile, the corresponding Meyer–Schuster products [49–54], first observed by Utimoto [49], were selectively obtained as illustrated with the formation of compounds **9** and **11** (Scheme 5).

**Scheme 6:**
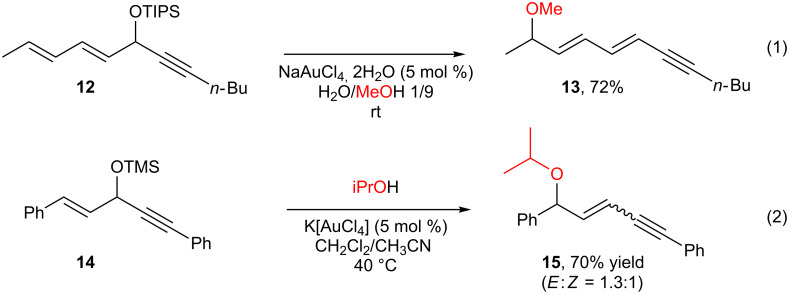
Silyl-protected propargyl alcohols in propargylic substitutions.

Interestingly, lowering the catalyst loading to 1 mol %, the substitution product **10** could be isolated in 60% yield (along with 35% of the Meyer–Schuster product **11**). This result could be explained by considering that **11** is derived from **10** through the conjugated addition of water (residual or produced during the substitution reaction) and a lower amount of gold catalyst should slow down the Meyer–Schuster process. It also suggests that, not only the OH group but also the OEt group can act as a good leaving group in these reactions. Indeed, when isolated compound **10** was subjected to Au(III) catalyst in the presence of water, **11** was obtained as the major product along with some unidentified by-products. Two consequences from these results are i) the use of an alcohol as nucleophile gives a product that can act as a substrate for further transformations (such as a Meyer–Schuster reaction), and ii) other ether (O–Si, O–C) functions can be used in these reactions. Indeed, the use of silyl protected OH groups is possible as illustrated by our group (Scheme 6, reaction 1) and by the Kirsch group (Scheme 6, reaction 2) [24,55]. In both cases selective S_N_’ reactions were observed, not only with oxygen nucleophiles but also with nitrogen and electron-rich aromatic nucleophiles [55]. It is worth noting that a mixture of S_N_ and S_N_’ products was obtained when allylsilane was used as the nucleophile with related substrates (Scheme 3).

**Scheme 7:**
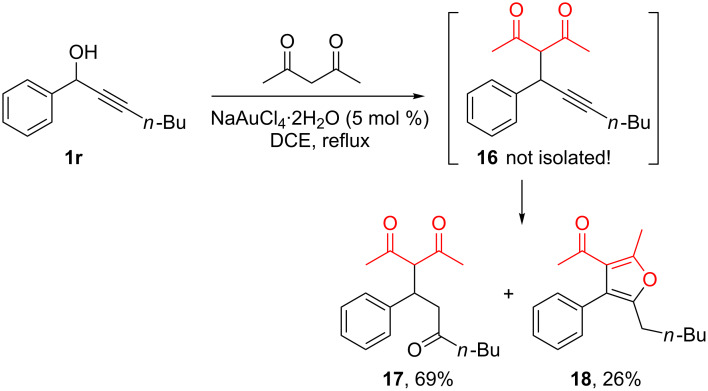
Acetylacetone as nucleophile in direct propargylic substitution.

When acetylacetone was used as the nucleophile, no reaction occurred with **1r** at room temperature, whereas in refluxing dichloroethane the substitution product **16** led to a mixture of products via triple bond hydration to give **17** [49–50,56] and furan formation to afford **18** (Scheme 7). The furan may possibly result from intramolecular addition of the enol form of acetylacetone. Related experiments have been independently reported by Arcadi [57].

The main limitations of the methodology appeared when azido (TMSN_3_), amides and phosphorus centered nucleophiles were used as nucleophiles: In these cases, either decomposition products or dimerization to the ether product were observed [58].

**Scheme 8:**
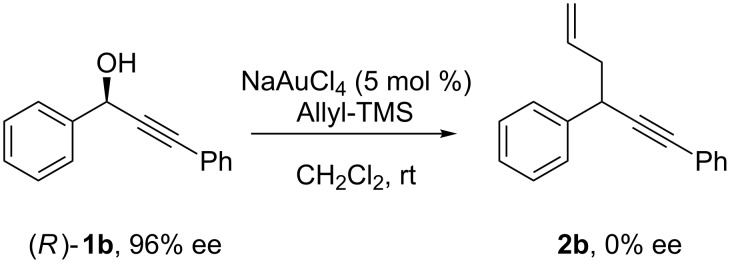
Enantiomerically enriched propargylic alcohols.

As previously noted, the lack of reactivity when aryl substituents bearing electron-withdrawing groups are present either at the propargylic or acetylenic positions, or when the propargylic position is only substituted by one alkyl group, suggests that an S_N_1-type reaction is involved. To confirm this hypothesis, the reaction starting from enantiomerically pure **1b** was carried out and the resulting substitution product **2b** was shown to be the racemate (Scheme 8).

**Scheme 9:**
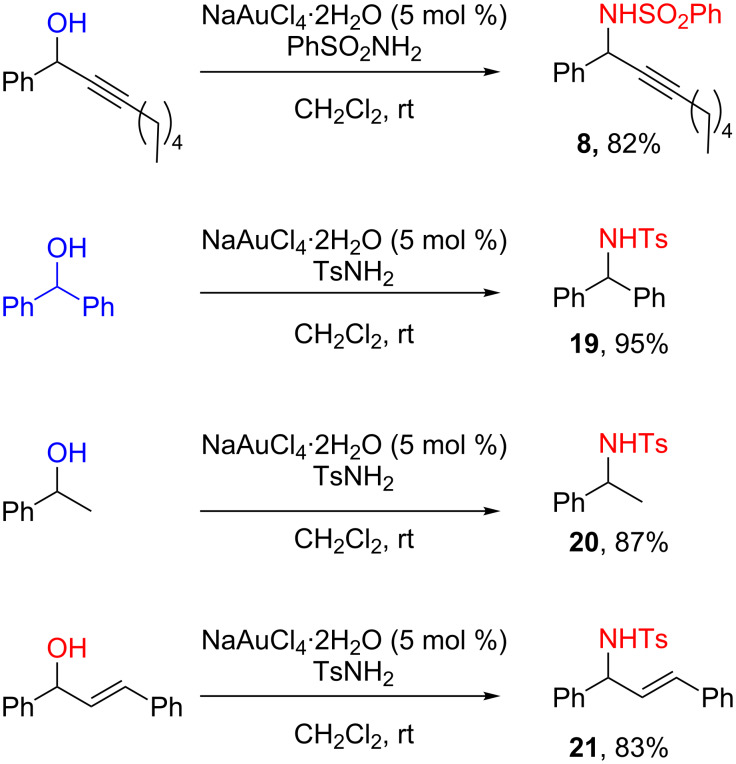
Scope of ‘activated’ alcohols in direct substitution reactions.

This result showed that the presence of triple bond may be not necessary and prompted us to extend the methodology to other activated alcohols such as diarylmethanol, benzylic and allylic alcohols (Scheme 9) [24,59–61]. This reaction was further extended to tertiary alcohols by Asensio [62].

**Scheme 10:**
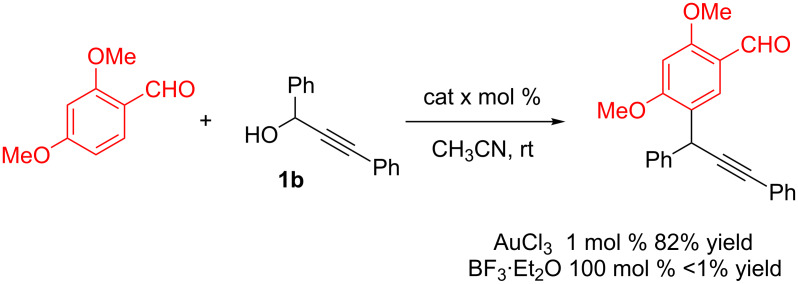
BF_3_ vs AuCl_3_ in propargylic substitutions [25].

Shortly after the publication of our seminal results [23], the direct substitution of activated (propargylic, benzylic and allylic) alcohols became of increasing interest and many efficient alternative methods were proposed [40–43]. Among these, great interest has been shown in the use of APTS or iron(III) catalysts due to their low cost and toxicity [26–29]. Despite its high cost, gold catalysis has some intrinsic benefits compared to other reported methodologies. Firstly, these Au(III)-catalyzed substitutions are clean and efficient processes and are usually carried out at room temperature. This point was very nicely illustrated by Dyker: In the reaction of 2,4-dimethoxybenzaldehyde with propargylic alcohol **1b** (Scheme 10), no substitution occurred at room temperature with a stoichiometric amount of BF_3_·Et_2_O but was complete with only 1 mol % of AuCl_3_ as catalyst [25].

Secondly, gold(III) possesses π-acidic properties and is able to activate triple bonds towards the addition of nucleophiles. Thus different reactivities and selectivities might be expected under gold catalysis conditions. For example, it could be interesting to combine both Lewis and π-acidities to promote domino reactions [44–45].

#### Domino reactions in the presence of bi-nucleophiles

**Scheme 11:**
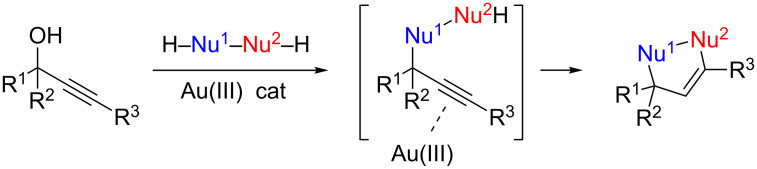
The use of bis-nucleophiles in direct propargylic substitutions.

We anticipated that by using bi-nucleophiles HNu^1^–Nu^2^H, the first nucleophilic substitution would be followed by activation of the triple bond and addition of the remaining nucleophilic group to the alkyne (Scheme 11) [63].

**Scheme 12:**
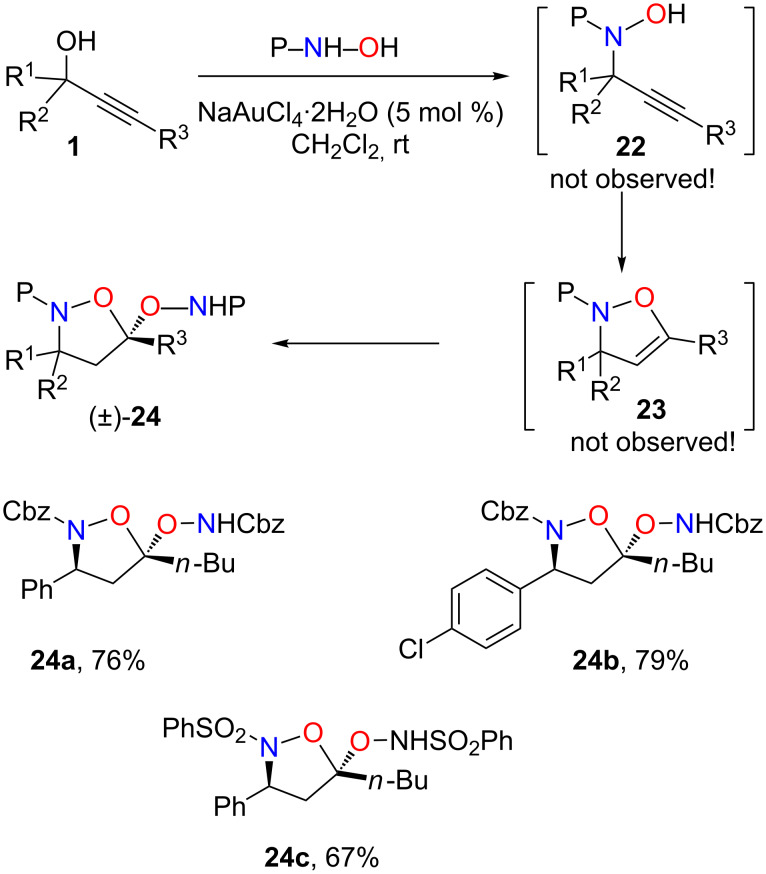
Tandem reactions from protected hydroxylamines and propargylic alcohols. P = Cbz, PhSO_2_.

Whereas no reaction occurred with bis-protected hydrazines, unexpected reactions occurred with protected (P = Cbz, PhSO_2_) hydroxylamines (Scheme 12). In model reactions of propargylic alcohol **1** with PhSO_2_NHOH in the presence of NaAuCl_4_ neither the propargylic substitution product **22** nor the expected isoxazoline **23** could be observed in the crude product. Instead a 1:1 mixture of unreacted alcohol **1** and compound **24** formally resulting from the addition of a second equivalent of hydroxylamine was obtained. By using 2.1 equivalents of the Cbz- or PhSO_2_-protected hydroxylamines, compounds **24a**–**c** were isolated in 67–79% yields as single diastereomers. Determination of the structures of **24** was a challenging task and could only be determined by an X-ray crystal structure determination on compound **24b** [63].

**Scheme 13:**
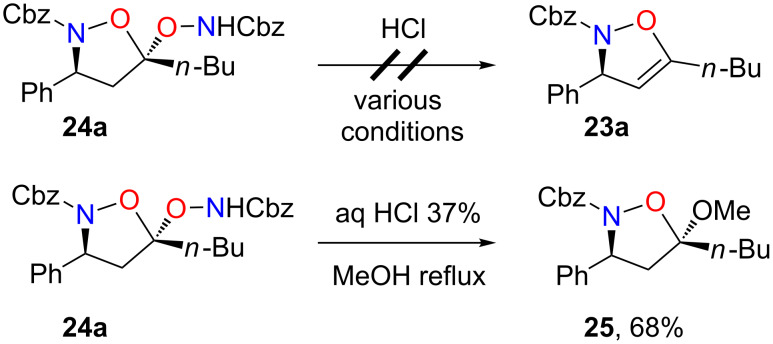
Tentative hydrolysis of bis-adduct **24a**.

Efforts to regenerate isoxazolines **23a** from **24a** by acidic treatment, under various conditions, were unsuccessful in our hands. Notably, in the presence of 37% aqueous HCl in refluxing methanol, the transacetalized product **25** was isolated in 68% yield (Scheme 13).

The desired isoxazolines cannot be directly obtained from propargylic alcohols **1** in the presence of gold(III) catalysts. From these preliminary experiments, it appears that the addition of hydroxylamine to the isoxazoline double bond is much faster than propargylic substitution. Consequently, the only possibility to obtain these isoxazolines would be to realize the propargylic substitution first of all (and consume all of the hydroxylamine) and subsequently perform the cyclization. Since iron(III) was known to be inefficient in the hydration of triple bonds [64–65], we anticipated that its use would lead only to the propargylic substitution product [28–29], and then, by adding a gold(III) catalyst, the cyclization could occur to yield the isoxazolines.

**Scheme 14:**
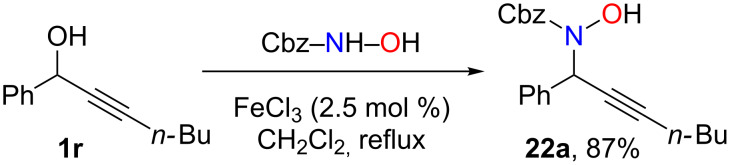
Iron-catalyzed propargylic substitutions.

Indeed, in the presence of iron(III) chloride [66–69], the substitution product **22a** was obtained in 87% yield, with no trace of the cyclized product even after prolonged reaction at reflux (Scheme 14). Very recently, Darcel described the iron(III) hydration of terminal alkynes [65]. These conditions were attempted to cyclize **22a** but in our hands only extensive decomposition was observed. We thus turned our attention to gold-catalyzed cyclization of **22a** which proved more difficult than initially expected (Table 2). As shown in Table 2, different metal salts were tried, and it was found that the best conditions were obtained when gold(III) was used in the presence of a catalytic amount (30 mol %) of DMAP [70].

**Table 2:** Propargylic *N*-hydroxylamine cyclization.

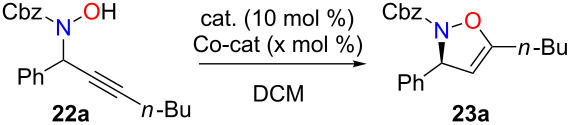				

Entry	Cat. (10 mol %)	Co-Cat mol %	*T*	Yield (%)

1	NaAuCl_4_·2H_2_O	—	rt or reflux	<20%
2	Ph_3_PAuCl	—	rt or reflux	—
3	Ph_3_PAuCl/AgOTf	—	reflux	8
4	ZnI_2_	DMAP 30	rt or reflux	15–20
5	FeCl_3_	DMAP 30	reflux	—
6	NaAuCl_4_·2H_2_O	DMAP 30	reflux	84

**Scheme 15:**
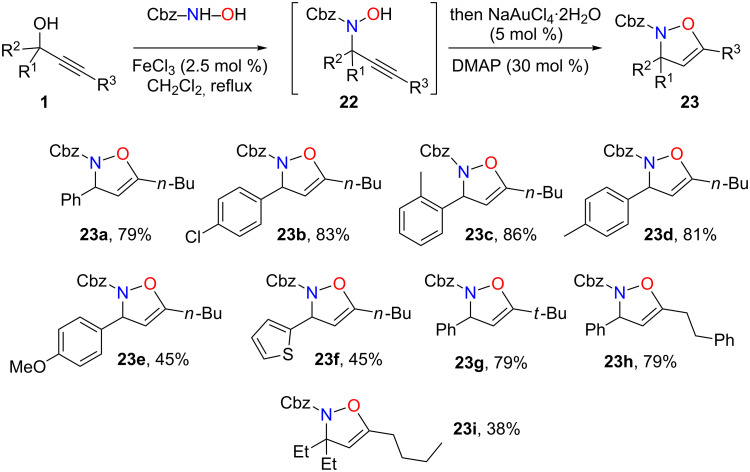
Isoxazolines formation.

We next tried to develop one-pot conditions for the direct transformation of propargylic alcohols **1** to isoxazolines **23**. Thus, treatment of the propargylic alcohol first of all with FeCl_3_ to promote the propargylic substitution followed by reaction with NaAuCl_4_, in the presence of DMAP 30 mol %, for the cyclization step as outlined in Scheme 15 proved to be efficient and compounds **23a**–**i** were obtained in 38–86% yield (Scheme 15).

**Scheme 16:**
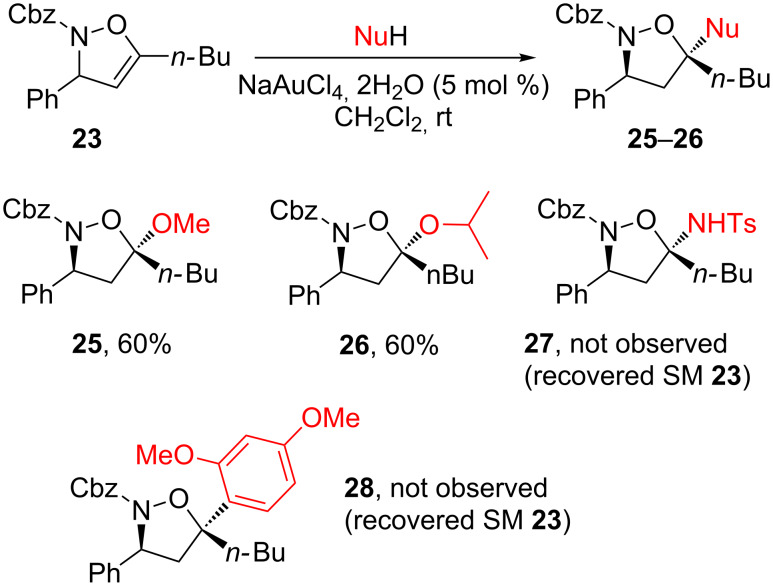
Addition of nucleophiles to isoxazolines.

Prompted by the apparently simplicity of the addition of hydroxylamine to the isoxazoline double bond (Scheme 12), we next tried to promote the gold-catalyzed addition of various nucleophiles. As illustrated in Scheme 16, the reaction proved restricted to alcohols and no reaction occurred with TsNH_2_ or electron-rich aromatic compounds such as 1,3-dimethoxybenzene.

**Scheme 17:**
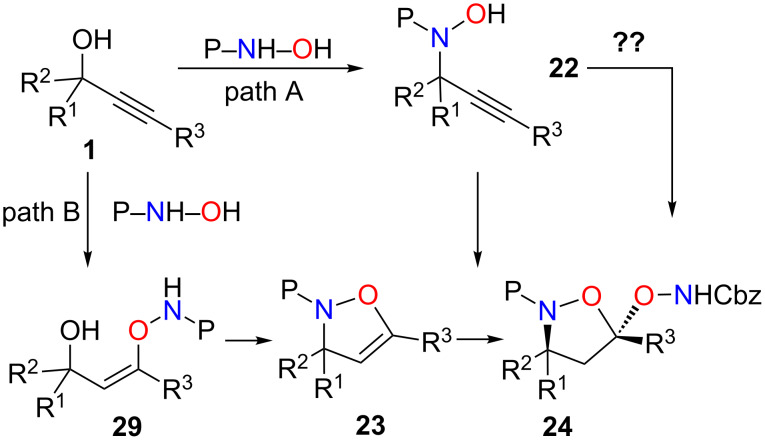
Potential mechanistic pathways.

Some contradictions arose from these experiments. On one hand, when propargylic alcohols **1** and protected hydroxylamines were treated with gold catalyst at room temperature (Scheme 12), the reactions were rapid and led to the formation of **24**. On the other hand, when trying to perform gold-catalyzed cyclization of the isolated propargylic substitution product **22**, the reaction appeared to be difficult and required the addition of a co-catalytic amount of DMAP, under reflux conditions, to be effective. Moreover, it has been shown that oxygen nucleophiles are prone to attack at the acetylenic site. Thus the reaction pathway could proceed in one of two ways: First, propargylic substitution by the nitrogen of the nucleophile followed by the cyclization (Scheme 17, path A) or attack on the acetylenic position by the oxygen of the nucleophile followed by intramolecular propargylic substitution (Scheme 17, path B).

**Scheme 18:**
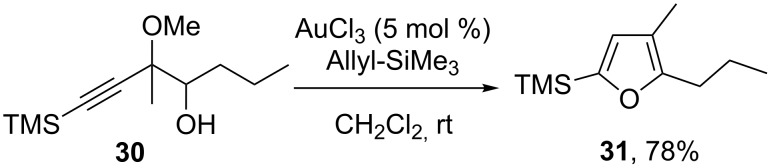
Synthesis of furans from homoproargylic alcohols.

The determination of the reaction pathway is a challenging task and to gain more insight we designed a model substrate **30** bearing a methoxy group in a quaternary substituted propargylic position and a (nucleophilic) alcohol function in the homopropargylic position. In order to deactivate the triple bond toward nucleophilic addition, a TMS group was placed in the acetylenic position. This substrate, obtained from the addition of an allenyl(cyano)cuprate on butyraldehyde [71–73], was reacted with allyltrimethylsilane. The reaction was totally regioselective for the propargylic site. Different reaction paths can be expected: i) The formation of a propargylic carbocation that can be attacked by allyltrimethylsilane or vicinal hydroxy group and ii) homopropargylic alcohol attack on the triple bond. The only product that could be isolated was the furan **31** which was obtained in 78% yield, with no trace of any product arising from an allylsilane attack (Scheme 18). The formation of the furan might be explained by a direct attack of the homopropargylic alcohol on the triple bond or a gold-catalyzed rearrangement of a transient epoxide as recently described by Blanc and Pale [74–76].

#### Synthetic developments

**Scheme 19:**
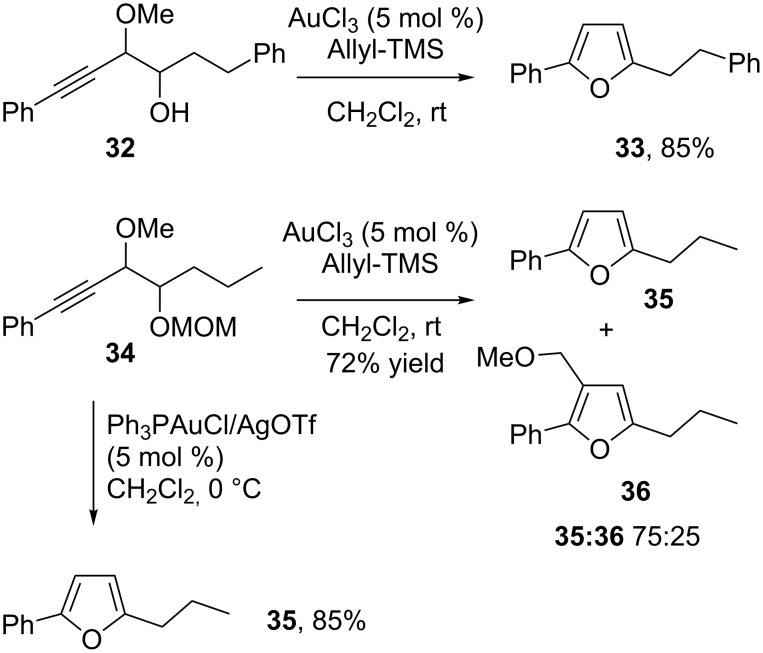
Synthesis of furans.

This result prompted us to explore the synthesis of functionalized furans from various homopropargylic alcohols obtained on a multi-gram scale, by our recently described allenylcuprate chemistry [71–73]. In the meantime, Aponick [77] and Akai [78] described very similar results using Au(I)/Ag(I) catalysts. Some of our gold(III)-catalyzed reactions are illustrated in Scheme 19. Interestingly, when the homopropargylic alcohol is protected as a MOM ether, a mixture of furans resulting from proto-demetallation **35** and MOM transfer **36** was obtained in a 75:25 ratio, respectively, whereas in the presence of gold(I) catalyst the proto-demetallation product **35** was the sole product. For related cyclization with subsequent migration of the ether substituent, see [79] and references therein. This experiment further highlights the strong propensity of oxygen nucleophiles for the addition to gold activated triple bonds.

**Scheme 20:**
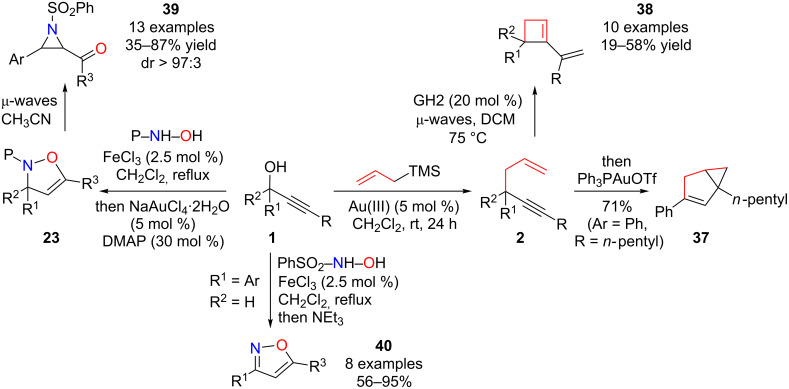
Propargylic substitutions: Synthetic applications. GH2 = Grubbs–Hoveyda 2^nd^ generation catalyst.

From allylated substitution products **2**, we were also able to develop two synthetic applications. In an one-pot, sequential, reaction with first a gold(III)-catalyzed propargylic substitution followed by a gold(I)-catalyzed cycloisomerization, the bicyclic compound **37** was obtained in 71% yield [24,80–82]. Very recently, a remarkable one-pot reaction using an original gold(III) catalyst has been described by Fairlamb [82]. We were also able, from compound **2**, to develop a 1,5-enyne metathesis that leads to functionalized cyclobutenes **38** (Scheme 20) [83], which was subsequently nicely illustrated by Goess in a total synthesis of grandisol [84].

From isoxazolines **23**, we were also able to promote, under micro-waves irradiation, a Baldwin rearrangement to yield the *cis*-acylaziridines **39**, with high diastereoselectivity [85]. Finally, from propargylic alcohols **1**, a sequential iron(III)-catalyzed propargylic substitution [28–29] followed by a NEt_3_-mediated elimination of the sulfonyl group and oxime cyclization gave, in a *one-pot* sequence, the corresponding isoxazoles **40** in good yields (Scheme 20) [86].

## Conclusion

In conclusion, we have developed gold(III)-catalyzed direct propargylic (allylic, benzylic) substitutions which have proved efficient with a great number of nucleophiles under mild conditions (dichloromethane, room temperature). A notable limitation of this methodology is since a stabilized positive charge is involved the scope of the reaction is limited to the use of tertiary or benzylic (allylic) propargylic alcohols. On the other hand, such a mechanism also implies that in the presence of chiral gold catalyst, through coordination to the triple bond (Scheme 1), enantioselective transformations could be obtained. We also found that with chiral gold(III) complexes very deceiving results can be obtained. It is worth noting that, recently, Bandini described very impressive enantioselective intramolecular direct allylic substitutions using chiral gold(I) complexes [87–88]. We also took advantage of the π- and σ-(Lewis) acidities of gold(III) complexes to promote domino reactions with bi-nucleophiles such as protected hydroxylamines. In the presence of gold(III), the expected isoxazolines **23** could not be directly obtained but required a dual iron(III)/gold(III) catalysis to be effective. Nevertheless, gold catalysts exert interesting π- and σ-Lewis properties, allowing direct nucleophilic substitution of various di or tri-substituted alcohols and cyclization reactions under mild conditions. This methodology gives easy access to various elaborated molecules with varied functionalities, as illustrated in this account.
